# Risk Mitigation of a Heritage Bridge Using Noninvasive Sensors

**DOI:** 10.3390/s25123727

**Published:** 2025-06-14

**Authors:** Ricky W. K. Chan, Takahiro Iwata

**Affiliations:** 1 School of Engineering, RMIT University, Melbourne 3000, Australia; 2 Department of Civil Engineering, Aichi Institute of Technology, Toyota 470-0392, Japan; t-iwata@aitech.ac.jp

**Keywords:** heritage bridge, reinforced concrete bridge, ambient vibration test, noninvasive sensors, digital twin, incident response framework

## Abstract

Bridges are fundamental components of transportation infrastructure, facilitating the efficient movement of people and goods. However, the conservation of heritage bridges introduces additional challenges, encompassing environmental, social, cultural, and economic dimensions of sustainability. This study investigates risk mitigation strategies for a heritage-listed, 120-year-old reinforced concrete bridge in Australia—one of the nation’s earliest examples of reinforced concrete construction, which remains operational today. The structure faces multiple risks, including passage of overweight vehicles, environmental degradation, progressive crack development due to traffic loading, and potential foundation scouring from an adjacent stream. Due to the heritage status and associated legal constraints, only non-invasive testing methods were employed. Ambient vibration testing was conducted to identify the bridge’s dynamic characteristics under normal traffic conditions, complemented by non-contact displacement monitoring using laser distance sensors. A digital twin structural model was subsequently developed and validated against field data. This model enabled the execution of various “what-if” simulations, including passage of overweight vehicles and loss of foundation due to scouring, providing quantitative assessments of potential risk scenarios. Drawing on insights gained from the case study, the article proposes a six-phase Incident Response Framework tailored for heritage bridge management. This comprehensive framework incorporates remote sensing technologies for incident detection, digital twin-based structural assessment, damage containment and mitigation protocols, recovery planning, and documentation to prevent recurrence—thus supporting the long-term preservation and functionality of heritage bridge assets.

## 1. Introduction

Bridges are fundamental elements of transportation networks, providing critical connections that support the movement of pedestrians, vehicles, and rail systems. Over time, exposure to environmental factors—including precipitation, wind, temperature variations, water currents, and wave action—as well as repeated loading from vehicular traffic, leads to progressive structural deterioration. In Australia, bridges are typically designed for a service life of 100 years. To ensure structural performance and user safety throughout this period, regular inspection and maintenance are essential.

For reinforced concrete bridges, corrosion of embedded steel reinforcement is a principal mechanism of deterioration, often resulting in concrete cracking and spalling. Other commonly observed forms of degradation include bearing deformation or misalignment, prestress losses, shrinkage, and creep. These issues, if left unaddressed, can substantially impair the structural capacity and serviceability of bridge systems.

In addition to structural performance considerations, the preservation of heritage bridges plays a vital role in promoting cultural and environmental sustainability. These structures embody the historical and architectural legacy of a community, contributing to its cultural identity, social continuity, and sense of place. By maintaining and rehabilitating heritage bridges rather than opting for demolition and reconstruction, it is possible to achieve significant environmental and economic benefits. Adaptive reuse and preservation strategies reduce material consumption, minimize construction-related emissions, and extend the life cycle of existing infrastructure. Furthermore, the conservation of such assets allows future generations to engage with and appreciate their historical significance, thereby reinforcing the societal value of sustainable infrastructure management.

### 1.1. Assessment of Historical Structures in Literature

There exists a substantial body of research dedicated to the structural assessment of historical bridges and buildings, employing a range of sensing technologies and analytical methodologies. Valente et al. [1] deployed accelerometers to validate a finite-element (FE) model of a historical steel truss bridge spanning the Tua River in Portugal. Batar et al. [2] applied terrestrial laser scanning to generate a high-resolution point cloud model of the Ayvalıkemer (Sillyon) masonry arch bridge in Turkey, enabling accurate geometric representation. Alpaslan et al. [3] conducted dynamic testing and finite element modeling to evaluate the structural behavior of a historical stone arch bridge, also located in Turkey. Granata et al. [4] utilized ground-penetrating radar (geo-radar) to investigate the internal reinforcement configuration of a cantilever reinforced concrete bridge in Italy. Ademovic [5] conducted a comprehensive structural assessment of a reinforced concrete arch bridge in Bosnia and Herzegovina, incorporating thermal imaging, non-destructive testing (NDT), and static load testing using heavy trucks. Bayane and Brühwiler [6] employed acoustic emission monitoring and strain gauge instrumentation to assess a steel–concrete composite bridge in Switzerland.

On the other hand, an accurate measurement of vertical displacement in bridges remains a significant challenge, particularly for heritage structures where invasive techniques are not appropriate. In response, researchers have developed various non-contact monitoring systems, which are especially well-suited for heritage bridge assessment. Zhao et al. [7] proposed a laser spot recognition system for long-term displacement monitoring. Brown et al. [8] introduced a hybrid laser–video displacement sensor. Waqas et al. [9] developed a response-based method using laser displacement sensors to detect malfunctioning steel bridge bearings. Pan et al. [10] applied an off-axis digital image correlation (DIC) technique to measure vertical bridge deflections with high precision. The DIC technique was also used to monitor deformation and crack propagation in concrete structures [11]. Recent developments in image recognition and artificial intelligence have found applications in using computer vision to detect defects in pavements and bridges [12,13,14]. To assess the dynamic properties of bridges, innovative methods include using test vehicles [15], parked cars [16], mobility vehicles and bicycles [17], and scooters [18]. Recently, the extreme function theory for damage detection has been applied to a case study of structural health monitoring of the well-known I-40 bridge [19]. In addition, recent development and popularity of using drone-facilitated robotic bridge inspections [20,21].

Common strategies for the assessment of historical bridges include: (i) static load testing using heavy vehicles combined with instrumentation, such as strain gauges and displacement sensors to capture structural response; (ii) dynamic testing to extract natural frequencies and mode shapes for finite-element model calibration; (iii) point cloud technologies for capturing the complex geometry of structures, such as masonry arch bridges; (iv) non-destructive testing methods for evaluating material properties and identifying signs of deterioration; and (v) non-contact displacement monitoring techniques, including laser-based systems and digital image correlation, for tracking structural deformation over time.

### 1.2. Current Practices of Bridge Assessments in Australia

The structural integrity of bridges in Australia is maintained through routine inspections and continuous maintenance activities, which are essential for the early detection and remediation of deterioration. At present, the majority of inspections rely heavily on visual assessment techniques.

According to AS5100 Part 7 [22], bridge inspections in Australia are categorized into three hierarchical levels:

- Level 1 inspections involve routine visual assessments aimed at identifying irregularities or anomalies that may warrant further investigation. Common issues noted during these inspections include excessive cracking, corrosion of steel components, degradation of expansion joints, and bearing deformations. In the state of Victoria, Level 1 inspections are typically conducted biannually by trained inspectors, not necessarily professional engineers. Level 1 inspections are also conducted after major events, such as flooding or passage of overweight, overmass vehicles.
- Level 2 inspections provide more detailed and quantified information to support asset management and maintenance planning. These visual assessments evaluate the outcomes of past rehabilitation efforts, identify current maintenance requirements, and support the forecasting of future condition deterioration and budgetary needs. In Victoria, Level 2 inspections are generally conducted every 3–5 years by more experienced bridge inspectors, often under engineers’ supervision.
- Level 3 inspections consist of comprehensive structural investigations undertaken by qualified engineers. These may involve advanced techniques such as structural modelling, load testing, and both destructive and non-destructive testing methods. Level 3 inspections are typically initiated in response to findings from Level 1 or 2 assessments and must be conducted by structural engineers or technical specialists.

Inspections and maintenance of vehicular bridges are the responsibilities of the state road agencies. State agencies published the traffic volume (Annual Average Daily Traffic, AADT) and bridge ratings in the public domain. The maintenance and repair of heritage bridges in Victoria are governed by detailed technical guidelines aimed at preserving both structural integrity and historical significance. The Bridge Maintenance and Repair Manual [23] serves as a key reference, outlining appropriate interventions based on the type and severity of deterioration observed during inspections. Emphasis is placed on using materials and techniques that are sympathetic to the original construction, ensuring that essential repairs do not compromise heritage values. To optimize resource allocation, maintenance activities are prioritized using a structured decision-making framework that considers factors such as the bridge’s physical condition, heritage classification, functional importance within the transport network, and available funding. Furthermore, compliance with the Heritage Act 2017 [24] is mandated for all state-registered structures, obligating asset owners to uphold minimum standards for maintenance and repair. This legal framework ensures that neglect or inappropriate interventions do not lead to irreversible damage or loss of cultural value, and it provides a mechanism for enforcement through repair orders or penalties where necessary. Together, these measures support a balanced approach to infrastructure management, integrating technical performance requirements with heritage conservation objectives.

Despite these structured practices, incidents of bridge failures globally have highlighted limitations in conventional inspection regimes. As a result, there is increasing interest in Structural Health Monitoring (SHM) as a complementary or alternative strategy. SHM involves the deployment of sensor networks to monitor key structural parameters, such as vibrational responses, crack widths, and deflections, in real time. This continuous data stream enables prompt identification of structural anomalies, thereby enhancing the responsiveness and reliability of bridge maintenance programs.

## 2. Descriptions of Heritage RC Bridge in This Study

**Figure 1:**
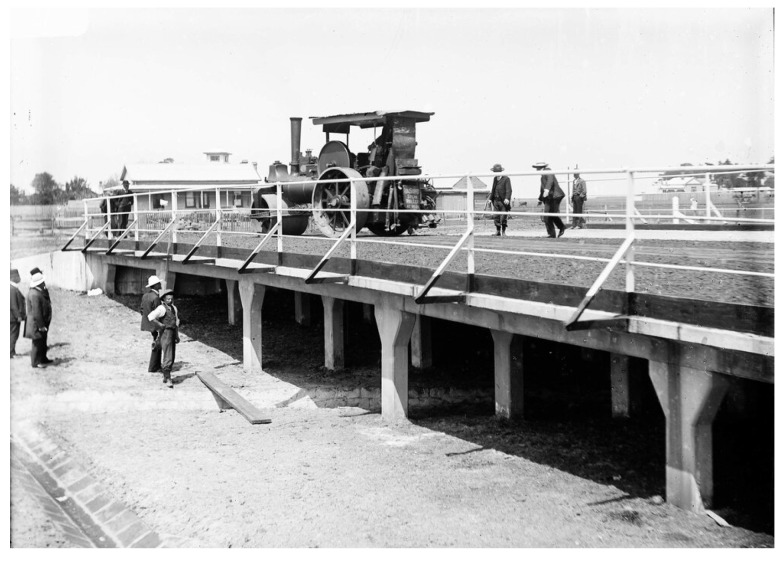
Load test conducted with a traction engine, circa 1905, University of Melbourne Archive (out of copyright).

The St Kilda Street Bridge (previously known as the Elwood Canal Bridge) is located approximately 10 km south of the Melbourne central business district, in the state of Victoria, Australia. According to the Heritage Council of Victoria Australia, the bridge possesses significant heritage value as it was the first reinforced concrete girder bridge in Victoria and, possibly, in Australia. It demonstrates the earliest stage in the development of reinforced concrete girders in the construction of bridges, a significant technological development of its time. It spans over a canal, which was a part of the drainage system in the low-lying swampy land in the Elsternwick and Elwood districts. The bridge was designed by renowned Australian engineer and military leader General Sir John Monash. Construction began in July 1905 and was completed in September of the same year. The construction of the bridge used the patented “Monier reinforced concrete method”. It utilized “compressive strength of concrete and tensile strength of iron”, which is essentially the modern-day principle of reinforced concrete. Figure 1 shows a photo of the load test conducted on the bridge with a traction engine.

**Figure 2:**
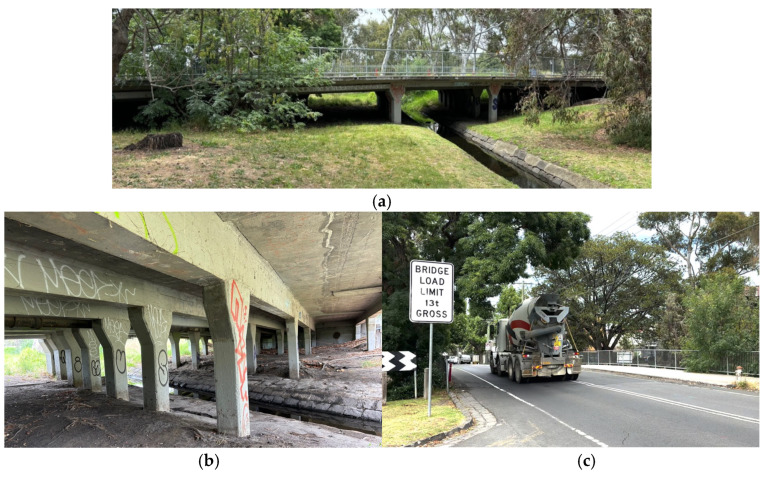
Current condition of bridge: (**a**) overview, (**b**) underside of bridge, and (**c**) road surface of bridge.

The bridge is a 5-equal-span, continuous reinforced concrete girder bridge with a 30-degree skew angle to suit local topography. The span between supporting columns is 20 feet (6.1 m). Total length was 100 feet (30.5 m), and the total width is 40 feet (12.2 m), consisting of two-way traffic roadways and a 10-foot (3.1 m)-wide footpath on one side only. There is no expansion joint between spans or at both abutments. The bridge is operational to the present time, and the current condition, 120 years after its construction, can be seen in Figure 2a–c. The reported Annual Average Daily Traffic (AADT) is 1300, with 7% truck traffic (year 2020 data) [25]. The road authority, Department of Transport, imposes a 13-ton gross limit on the bridge; signage can be seen in Figure 2c, although strict enforcement could not be observed. As can be seen in Figure 2c, a large, bi-axle concrete agitator truck with an estimated weight of 28 tons when fully loaded passed through the bridge, exceeding two times the mass limit imposed on the bridge. The bridge is protected under the Victorian Heritage Register, which provides legal protection for heritage buildings, bridges, archaeological sites, or other objects. The bridge cannot be altered in any way without an exemption permit. It includes any kind of retrofit work.

### Previous Studies of St Kilda Street Bridge

In early 2000, the bridge underwent a thorough structural assessment. The works included carbonation, as described in [26]. Their work consisted of standard in situ tests, such as cover thickness measurements, compressive tests of drilled cores, and corrosion rate measurements. Their work also included grillage analysis and load capacity assessment. They concluded that significant deficiencies were observed in both flexure and shear actions under the 68-ton HML B-double truck loading. In particular, the available load capacity factor for shearing action was as low as 0.62. Table 1 shows some of the parameters identified in this study. It should be noted that the tensile strengths of reinforcement were not investigated in their study.

In 2014, a structural assessment was conducted by a private engineering consultant firm, indicating the condition is “poor” [27]. Considerable spalling of concrete and loss of reinforcement were indicated by a visual inspection. The latest repair of the bridge was reported in March 2015 [28]. The latest bridge rating, determined by the Department of Transport of Victoria and published in the public domain, is 7, indicating the bridge is in excellent condition. However, it should be noted that this rating was assessed shortly after the structural refurbishment was completed in April 2015. There was no reported assessment after this date.

## 3. Field Inspections and Deployment of Sensors

### 3.1. Field Inspections

**Figure 3:**
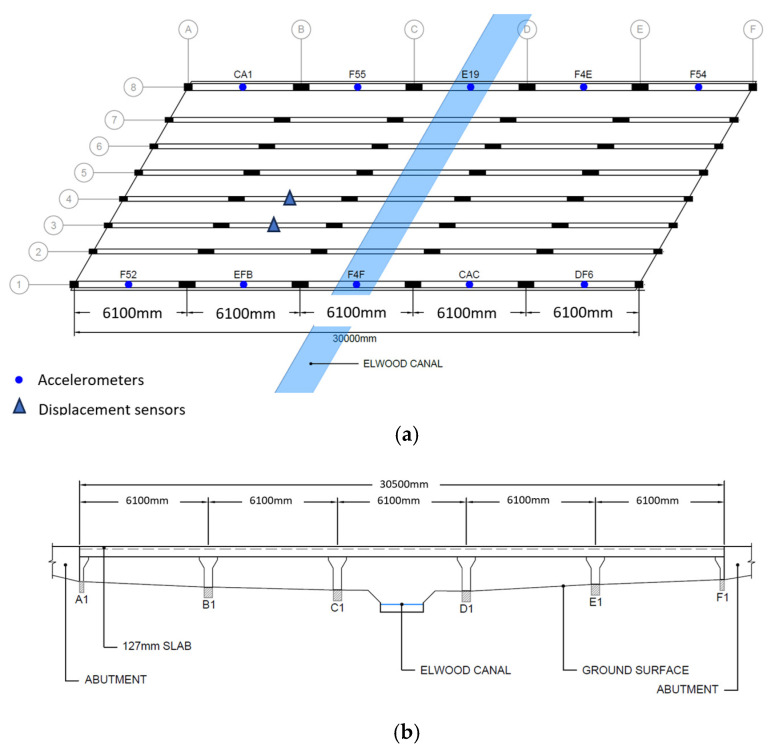
Bridge geometry and sensor placements: (**a**) plan view and (**b**) elevation view.

Visual inspections were conducted by the authors during periods of low water flow in the Elwood Canal, which facilitated convenient access to the underside of the bridge. This allowed for close-range examination of the bridge beams and deck soffit. In the absence of original design drawings, the bridge geometry was manually measured and is presented in Figure 3. Overall, the structure was found to be in satisfactory condition. Common durability issues, such as concrete spalling or exposed reinforcement, were not observed. Notably, defects reported in earlier condition assessments from 2003 and 2014, prior to the structural rehabilitation undertaken in 2015, were no longer evident. This suggests that the repair interventions performed in 2014 have remained effective, with no signs of delamination in the patched areas.

**Figure 4:**
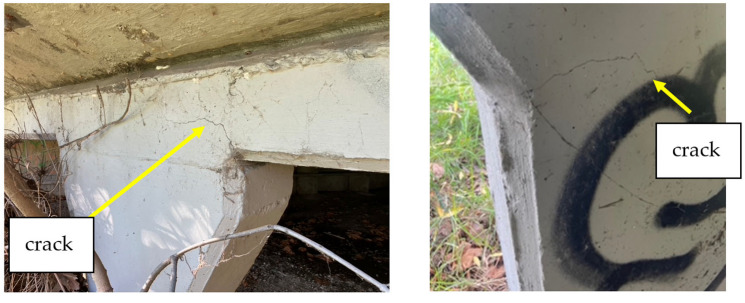
Shear cracks identified near bridge piers.

However, shear cracking was observed near the haunch regions of several columns, as illustrated in Figure 4. No evidence of scouring or erosion was found around the bridge piers, indicating that water flow at the site has not adversely affected the substructure. Surface asphalt was in good condition, and the conditions of handrails were satisfactory.

### 3.2. Ambient Vibration Test

The Ambient Vibration Test (AVT) is a non-destructive testing method used primarily in the field of civil and structural engineering. It involves measuring the natural vibrations of a structure (such as a building, bridge, or dam) caused by ambient environmental factors such as wind, traffic, and seismic activity. These vibrations occur at very low amplitudes and do not require the application of external force. The dynamic properties obtained from AVT in this study are used to validate the finite-element models of the structure in Section 4.

In this study of a heritage bridge, AVT was chosen for its particular suitability for a heritage bridge:

1. Non-destructive—it does not involve any coring or intrusive procedure. This is particularly important for heritage-listed assets.
2. Road closure was not required—the bridge has an AADT of 1300; closure of the bridge will adversely affect local residents.
3. No external application of force, such as an impact hammer, was required.
4. No external power source is required—sensors, the DAQ unit, and the computer have an internal battery.
5. It measures the structure’s response under actual service conditions.

**Figure 5:**
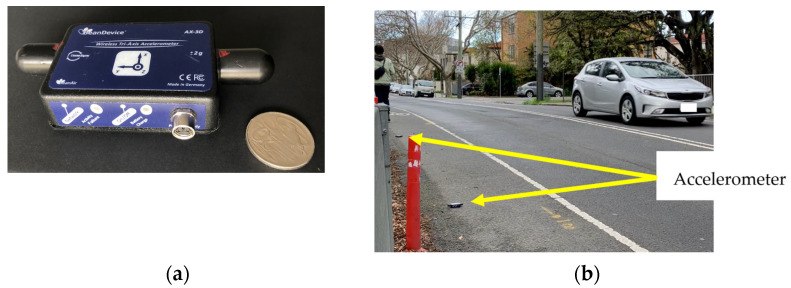
Ambient vibration test: (**a**) wireless accelerometer and (**b**) field deployment.

Ten wireless accelerometers, BeanAir BeanDevice 2.4 GHz AX-3 D (acquired in Melbourne, Australia), denoted as CA1, F55, E19, etc. in Figure 5, were evenly distributed on two sides of the bridge. Due to the heritage nature of the bridge, any intrusive fixture, such as bolting, was avoided. The sampling rate was set to 100 Hz. Data requisition was conducted over the software Beanscape (version 1.28.0.26), supplied by the same manufacturer. The software allows streaming data over the wireless network, as well as downloading data from the sensor’s internal storage.

**Figure 6:**
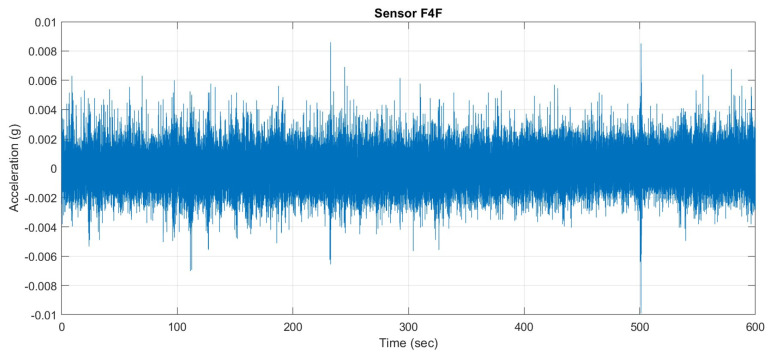
Typical time history of recorded vibrations.

**Figure 7:**
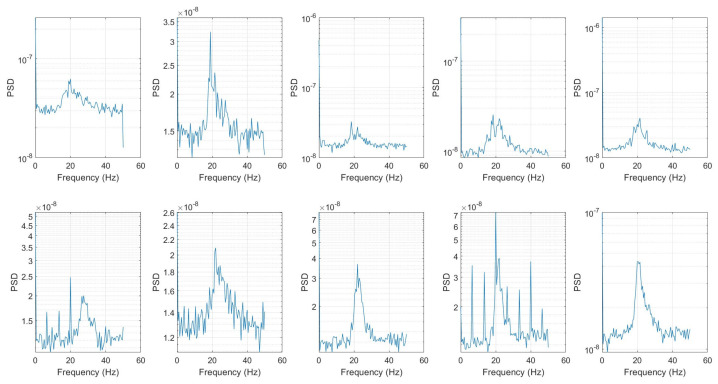
Power spectral density plots (figures arranged in accordance with placement in Figure 3).

Under ambient vibrations caused by passing traffic and environmental excitations, a typical time history of the sensor reading is shown in Figure 6. Power Spectral Densities (PSD) determined by the Welch method are shown in Figure 7. The PSD diagrams are arranged in the same order as sensor placement shown in Figure 3a for easy reference.

The PSD results show a common sharp peak at approximately 20 Hz, indicating that it is a strong candidate for the global fundamental frequency of the bridge. Higher vibration modes are likely to be higher than 50 Hz, which is higher than the Nyquist frequency, due to the limitation of the sampling rate. Sensor F52 and CAC detected several other peaks, but they are absent from other sensors. Local excitation, such as vehicle passing near those points or local structural features, could contribute to those peaks. Additionally, calculations were done to try to determine the mode shape for each mode based on the vibration data and natural frequencies. It should be noted that a higher sampling rate could be used in future testing to capture higher dynamic modes. Mode shape computation can be done in two ways: one uses both input and output data, while the other just uses the output vibration data. The ambient vibration test limited the available approach options to solely measure output vibration data. Using input and output data, the Frequency Response Function (FRF) can be used to derive the mode shape. Although this method offers great accuracy, the system needs input data. Getting the input data for an actual bridge with traffic is really challenging. Other computation techniques, such as the Eigensystem Realization Algorithm (ERA) [29], Frequency Domain Decomposition (FDD) [30], and Stochastic Subspace Identification (SSI) [31], can be used if input data are not available. ERA is a system identification method used to extract modal parameters from free vibration data. It is efficient, works with output-only data, and is widely used in structural dynamics to identify frequencies, mode shapes, and damping. The ERA approach was chosen for this study.

The ERA is a time-domain system identification method that uses impulse response data or an approximation to estimate the system’s modal parameters, including natural frequencies, damping ratios, and mode shapes, which are particularly suitable for linear time-invariant systems. Such a linear system is described by a state-space representation,

(1) $$x_{k+1}=Ax_{k}+Bu_{x}$$

(2) $$y_{k}=Cx_{k}+Du_{k}$$

where:

*x_k_* is the state vector, *u_k_* is the input vector, and *y_k_* is the output vector. *A*, *B*, *C*, and *D* are system matrices. An impulse input at time *k* = 0; the output becomes the Markov parameters:

(3) $$y_{k}=\left\{\begin{array}{c} D,\;\;\;\;\;\;\;\;\;\;\;\;\;\;k=0\;\\ CA^{k-1}B,\;\;\;\;\;\;\;\;\;\;\;\;\;k\geq 1\;\;\;\;\;\; \end{array}\right.$$

Defining a block Hankel matrix *H*_0_ using the measured output impulse responses:

(4) $$H_{0}=\left[\begin{array}{ccc} Y_{1} & \ldots & Y_{s}\\ \vdots & \ddots & \vdots \\ Y_{r} & \ldots & Y_{r+s-1} \end{array}\right]$$

where *r* and *s* are user-defined dimensions, which are large enough to capture system dynamics. Apply Singular Value Decomposition (SVD) to the Hankel matrix:

(5) $$H_{0}=U\Sigma V^{T}$$

where

*U* is the left singular vector

Σ = diagonal matrix of singular values

*V* = right singular vectors

Truncate to *n* dominant singular values where *n* is the system order

(6) $$H_{0}\approx U_{n}\Sigma_{n}V_{n}^{T}$$

The system matrix estimation can be conducted by forming the shifted Hankel matrix *H*_1_ (one step ahead in time) and computing

(7) $$A_{est}={\Sigma_{1}^{-1/2}U_{1}^{T}H}_{1}V_{1}\Sigma_{1}^{-1/2}$$

*A_est_* refers to the estimated state transition matrix. Eigen-decomposition of A is conducted by

(8) $$A_{est}\Phi =\Phi \Lambda$$

where

(9) $$\Lambda =diag(\lambda_{1},\lambda_{2},\ldots ,\lambda_{n})$$

and Φ is the eigenvector of A.

From *A_est_*, eigenvalues (natural frequencies and damping) and eigenvectors (mode shapes) can be computed. Let *λ_i_* be the eigenvalues of *A_est_*, and convert to continuous time using sampling period *T_s_*,

(10) $$s_{i}=\frac{ln(\lambda_{i})}{T_{s}}$$

Natural frequency can be obtained by

(11) $$f_{n}=\frac{\left|Im(s_{i})\right|}{2\pi }$$

Damping ratio

(12) $$\xi =-\frac{Re(s_{i})}{\left|s_{i}\right|}$$

### 3.3. Girder Displacements and Crack Widths Under Traffic Loads

**Figure 8:**
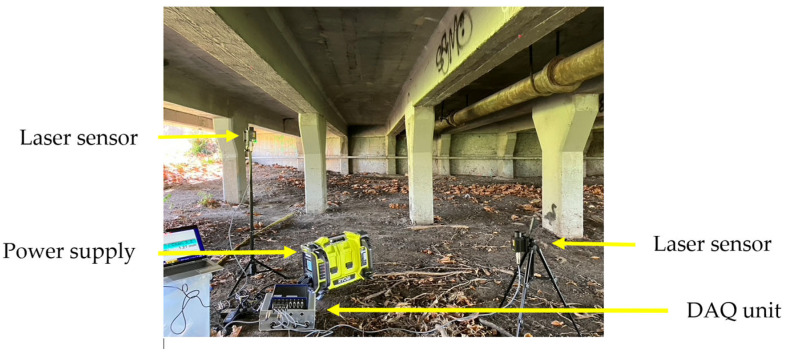
Non-contact displacement measurement by laser sensors.

**Figure 9:**
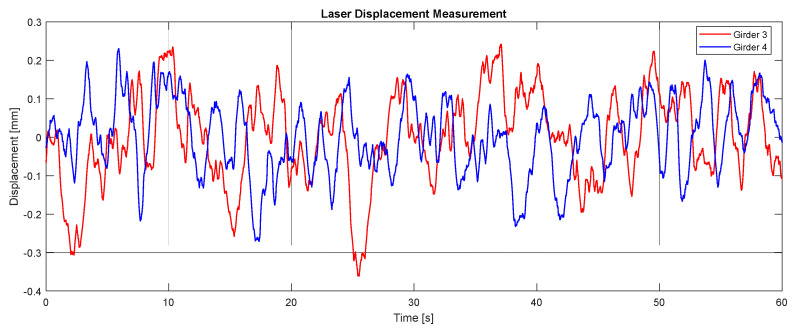
Typical displacement measurements.

The authors also conducted measurements to quantify girder displacements induced by traffic loading. For this purpose, non-contact laser displacement sensors were selected. Two OptoNCDTILR2250-100 sensors (resolution: 0.1 mm; range of sensors is 100 m, manufacturer: Micro-Epsilon, Melbourne, Australia) were installed at the mid-span locations of Girders 3 and 4. Data acquisition was facilitated using an 8-channel measurement amplifier (Me-Systeme GSV-8 DS, Melbourne, Australia), operating at a sampling frequency of 50 Hz. The experimental setup for displacement monitoring is depicted in Figure 8. Measurements were performed over a 20 min period during afternoon conditions with light traffic. A representative 60 s segment of recorded girder displacement is presented in Figure 9. Results indicated minor deflections, with maximum displacements remaining below 1 mm (code deflection limit is *L*/600 or 8.8 mm)

**Figure 10:**
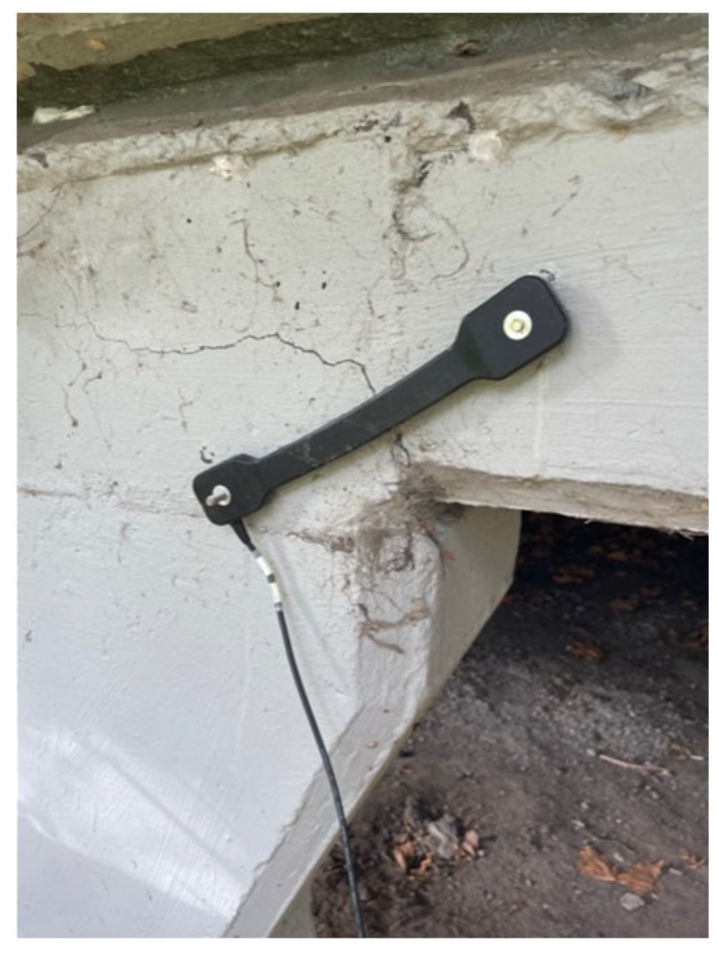
Crack width measurement with elastic displacement sensor.

In addition, an attempt was made to capture crack width variation under traffic-induced loading. An elastic displacement sensor (ElastiSense DS-100, Melbourne, Australia) was temporarily affixed perpendicular to a pre-identified crack, as shown in Figure 10. However, no measurable crack opening was observed during the testing period, likely due to the relatively low traffic loads encountered on the test day.

## 4. Development of Finite-Element Model

### 4.1. Finite-Element Modeling

A numerical model consisting of beam and plate elements was developed using the FE package Spacegass (version 14.2). The bridge deck was modeled as a plate element with a thickness of 127 mm (5 inches), which is consistent with field measurement and the previous report [27]. All bridge girder and pier sizes were measured in the field and replicated in the software. Pier supports are modeled as fixed-ended, and the plate boundary at the two ends of the bridge deck was modeled as pin-supported. The physical constraint of the abutment at the two ends of the bridge acted as translational restraints. The Young’s Modulus was taken as 22,500 MPa, according to [26].

**Figure 11:**
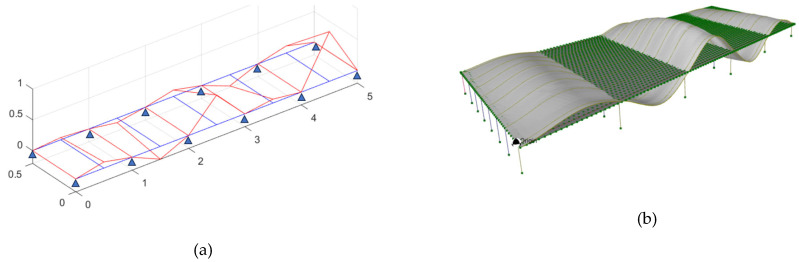
Fundamental mode shape of (**a**) Ambient Vibration Test and (**b**) FE model.

A dynamic frequency analysis was performed to evaluate the structural behavior of the bridge. Table 2 presents a comparison of the fundamental frequencies obtained from field measurements and the finite-element (FE) model. The corresponding mode shapes are illustrated in Figure 11. The results indicate that the FE model reliably captures the global flexural response of the structure without requiring further model calibration or updating. This agreement suggests that the assumed material properties, boundary conditions, and overall stiffness distribution in the FE model are representative of the actual bridge. However, due to limitations inherent in ambient vibration testing, higher-order vibration modes could not be clearly identified, restricting the assessment of complex dynamic characteristics. Despite this, the FE model effectively reflects the dominant structural behavior and is deemed suitable for conducting serviceability evaluations, including the parametric what-if scenario analyses presented in subsequent sections.

### 4.2. Scenario Analysis—Passage of Overweight Vehicles

In the event of an overweight vehicle traversing the bridge, structural elements may be subjected to excessive forces that exceed their design capacity, potentially leading to irreversible damage such as cracking. Due to the absence of detailed reinforcement information and precise material properties for this heritage structure, a rigorous capacity assessment cannot be performed with high confidence. Therefore, a comparative analysis is adopted to illustrate the potential impact of overweight vehicle loading.

**Figure 12:**
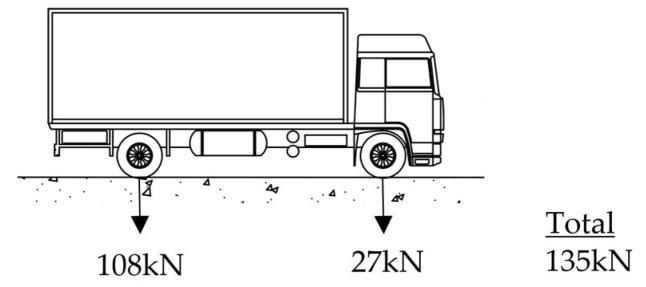
M13.5(H15) vehicle load to AASHTO.

The current posted load limit for the bridge, as designated by the local road authority, is 13 tonnes (127.5 kN). This value closely corresponds to the two-axle M13.5 vehicle load defined in the AASHTO Standard Specifications for Highway Bridges [32], as illustrated in Figure 12. For the purposes of this study, the M13.5 vehicle is considered representative of the allowable safe load.

To assess the effect of overweight vehicles, structural actions induced by two representative rating vehicles—namely the T44 (44-tonne semi-trailer truck) defined in the 1992 Austroads Bridge Design Code [33], and the M1600 vehicle specified in the latest AS5100.2–2017 [34] —are compared against those induced by the M13.5 vehicle. Analyses were conducted in three potential lanes: the northbound lane, the southbound lane, and in the middle of the bridge. No lane factor or dynamic load factor was applied for comparative study. This approach enables a qualitative evaluation of the increased demand imposed by heavier vehicles on the structural system, providing insight into the potential risks associated with overloading. Results are shown in Table 3.

The simulation results indicate that the T44 truck induces up to a 68% increase in shear force and an 81% increase in bending moment in the bridge girders when compared to the baseline M13.5 vehicle. The M1600 vehicle imposes an even more severe demand, generating shear forces and bending moments exceeding three times those of the allowable load case. On the other hand, deflection estimation using clear span *L*=5255 mm and serviceability vehicle loads indicated that deflections caused by these design vehicles were all well under the code limit of *L*/600. This was primarily due to the very short span of this heritage bridge. As such, the main consideration is that the significant increases in internal forces due to shear and moment caused by overweight vehicles are likely to cause cracking or, in extreme cases, structural failure of the bridge girders. These findings underscore the critical importance of strict enforcement of the posted weight limit to preserve the structural integrity of this heritage bridge.

**Figure 13:**
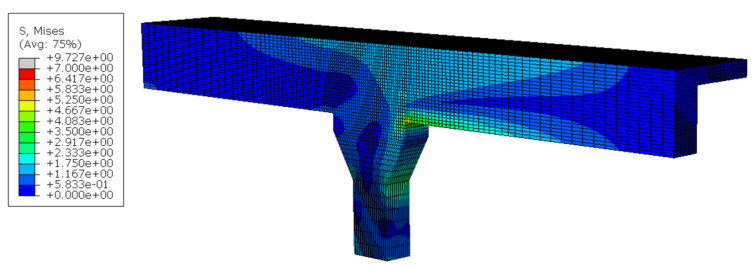
Stress contour of detailed FE modeling.

**Figure 14:**
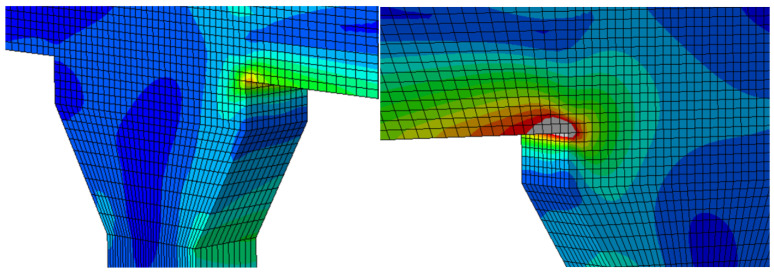
Stress concentrations at girder–pier junction.

A supplementary investigation into the effects of overloading was conducted using a nonlinear finite-element model developed in Abaqus, employing the Concrete Smeared Cracking material model. The analytical model comprised a representative girder–pier sub-frame, in which the base and one girder end were fixed, while a prescribed displacement, equivalent to the mid-span displacement due to an M1600 vehicle, was applied to the opposite end of the girder. The resulting stress distribution is illustrated in Figure 13 and Figure 14. Notably, stress concentrations were observed at the girder–pier interface and at the transition region where the pier cross-section widens. These localized stress zones align well with the locations of visible cracking identified during the field inspection (refer to Figure 4), thereby validating the model’s predictive capability.

### 4.3. Scenario Analysis Loss of Foundation Due to Scouring

**Figure 15:**
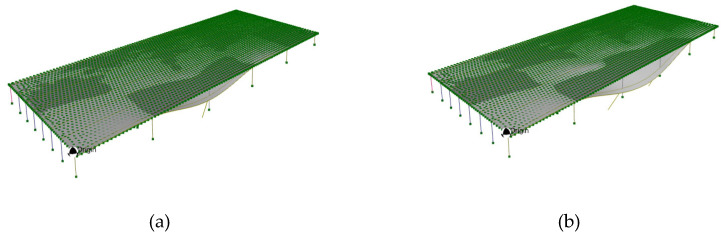
(**a**) Fundamental mode shape with Pier C1 scoured; (**b**) fundamental mode shape with Pier C1 and D1 scoured.

This section investigates the potential influence of scour damage at one or more bridge piers on the dynamic characteristics of the structure. Specifically, it aims to examine whether such damage would manifest as measurable changes in the identified mode shapes and corresponding natural frequencies obtained through ambient vibration testing. To simulate the effects of scour, structural damage was introduced in the finite-element model by removing translational restraints at piers C1 and D1, thereby representing a loss of lateral and vertical support due to foundation instability. Piers C1 and D1 were selected for this simulation based on their direct exposure to the predominant river flow, which increases their vulnerability to scour. Scour damage is particularly challenging to detect through routine bridge inspections, as it typically occurs below the waterline and remains hidden from visual assessment [35,36]. If scour-induced degradation leads to detectable shifts in modal frequencies or alterations in mode shapes, ambient vibration-based modal analysis may offer a practical and non-invasive means for early detection and monitoring of scour-related damage in bridge foundations. Figure 15 shows the fundamental mode shapes of the two scour scenarios and Table 4 illustrated the change in fundamental frequencies.

The simulation results suggest that foundation instability due to scour can lead to significant shifts in the natural frequencies and notable alterations in the mode shapes of the bridge. These findings highlight the potential utility of routine ambient vibration testing as a non-invasive tool for monitoring changes in the bridge’s dynamic response, thereby enabling early detection of scour-related damage.

## 5. Proposed Incident Response Framework for Heritage Bridges

**Figure 16:**
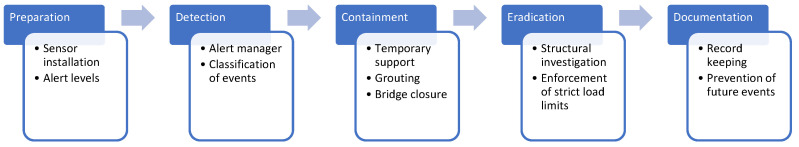
Proposed Incident Response Framework (IRF) for heritage bridges.

Heritage bridges represent critical historical assets with considerable social and engineering significance. However, they are frequently subject to various forms of deterioration and typically exhibit lower structural capacity when compared to contemporary bridge designs. As such, they necessitate a distinct and heightened level of attention in their management and preservation. Based on insights gained from the structural assessment of the St Kilda Street Bridge presented in this study, an Incident Response Framework (IRF) has been developed, specifically designed to address the unique needs of heritage bridge structures. The proposed IRF comprises five sequential stages intended to prevent inadvertent damage, mitigate consequences in the event of an incident, and minimize the risk of recurrence. A simplified schematic of the six-stage IRF is illustrated in Figure 16. It is important to emphasize that the framework is currently a conceptual proposal and does not represent existing procedures or policies implemented by the relevant road authority.

### 5.1. Phase 1—Preparation

The preparation phase entails the strategic planning and deployment of Structural Health Monitoring (SHM) systems on heritage bridge structures. This includes the selection and installation of sensors such as strain gauges, displacement sensors, and accelerometers, which are tailored to the structural configuration and material composition of the bridge. Recent advancements have made both wireless and wired sensors widely accessible, enabling real-time data acquisition and continuous remote monitoring of key structural parameters [37]. For heritage bridges, non-invasive sensors, such as non-contact laser displacement sensors, should be selected.

Effective monitoring of structural conditions relies on the acquisition of high-quality, real-time measurements from critical structural components. These data are transmitted to control systems that can trigger alerts upon detection of anomalous behavior, thereby facilitating timely interventions. SHM systems enhance the capacity of maintenance personnel to make informed decisions regarding asset management, ultimately contributing to public safety. A fundamental SHM setup typically comprises a network of sensors capable of capturing and analyzing variables such as stress, strain, vibration, tilt, and temperature, in accordance with established specifications, performance criteria, and environmental conditions. A wealth of knowledge in terms of sensor selection can be found in the literature [38,39].

### 5.2. Phase 2—Incident Detection and Classification

In this phase, the Structural Health Monitoring (SHM) system initiates an alert upon the detection of structural anomalies or deviations from normal operating conditions. Following the alert, the incident response team proceeds to classify the event based on predefined criteria into one of four categories: Minor, Moderate, Major, or Emergency. This classification facilitates the formulation of an appropriate and proportionate response strategy.

- Minor Incident: Events that exceed the established benchmark but remain within 50% above the permissible threshold. Typical examples include superficial issues such as hairline cracks or localized surface corrosion on steel components.
- Moderate Incident: Events that surpass the minor incident threshold (i.e., more than 50% above the benchmark) but remain under 75% of the allowable limit. Examples include noticeable girder deflections beyond tolerance, non-critical foundation settlements, or progressive cracking in the deck slab.
- Major Incident: Events that exceed 75% of the permissible limit. These include critical issues such as bearing misalignments, partial failures of girders or pier caps, or extensive corrosion and cracking that may compromise structural integrity.
- Emergency Incident: Severe, sudden, and unanticipated events that significantly exceed allowable thresholds and impair the functionality of the bridge. Examples include natural disasters (e.g., earthquakes, cyclones) or catastrophic impacts (e.g., vehicle or aircraft collision), which necessitate the immediate suspension of all structural use to prevent potential collapse.

To support reliable classification, clear benchmarks must be established, with well-defined thresholds and diagnostic rules that differentiate each incident category based on quantitative limits and observed conditions. The parameters for incident classification are:

- Minor: >Benchmark but ≤50% of permissible limit
- Moderate: >50% but ≤75% of permissible limit
- Major: >75% of permissible limit
- Emergency: Far exceeds permissible limits; sudden and disruptive

Following classification, the response team evaluates the severity and scope of the event, collects supplementary evidence, and performs root cause analysis to inform further action and mitigation strategies.

### 5.3. Phase 3: Containment

Following the detection and classification of an incident, the subsequent phase involves deploying a team of structural engineers and/or bridge inspectors to undertake detailed visual assessments and to develop both short- and long-term containment strategies. The primary objective of this phase is to mitigate further deterioration and to stabilize the affected structural elements.

Short-Term Containment: Measures are implemented in response to minor incidents that can be addressed without interrupting the normal operation of the bridge. These measures are intended to maintain the structure’s usability while preventing immediate escalation of damage. While such interventions restore short-term functionality and enhance safety, they do not constitute permanent repairs, and the affected components may remain vulnerable to progressive deterioration.

Example: An overweight vehicle traversing the bridge may induce excessive bending in the girders and deck slab, resulting in the formation of microcracks or hairline cracks at the tension zones of the girders. Although these cracks may not immediately compromise structural integrity, repeated loading could propagate the damage, potentially leading to critical failure. To mitigate this risk without removing the bridge from service, crack filling using epoxy or cementitious grouting may be employed to restore local continuity and delay further degradation.

Long-Term Containment: Strategies involve the complete isolation of the structure from public use and the cessation of all traffic and operational activities. This approach is adopted when the damage poses a significant risk and requires comprehensive repair or retrofitting. Long-term interventions may include strengthening, replacement of damaged elements, or retrofitting measures aimed at restoring structural performance and ensuring long-term reliability.

Example: In the aftermath of a cyclone, if girders become displaced or unseated from their bearings, continued operation of the structure presents an unacceptable risk. Immediate closure is required, followed by detailed inspection and structural retrofits. The affected components—such as the bridge bearings, girders, and deck slab—must be lifted using cranes or hydraulic jacks, repositioned, and secured to their original alignments to reestablish stability and safety. Damaged components will need to be replaced.

### 5.4. Phase 4: Eradication

This phase focuses on a comprehensive analysis of data obtained from the Structural Health Monitoring (SHM) system to identify the root cause of the structural anomaly. A detailed evaluation of the recorded data facilitates a deeper understanding of the factors that contributed to the incident and supports the development of targeted mitigation strategies to prevent recurrence.

This process involves correlating SHM data with incident-specific observations to determine underlying failure mechanisms or operational deficiencies. Root cause identification is critical to inform the design of evidence-based interventions, ranging from policy updates to physical modifications, that enhance the long-term resilience of the structure.

Example: If the SHM system records indicate that microcracking occurred due to repeated instances of multiple overloaded trucks crossing the bridge simultaneously, regulatory measures can be introduced to control such loading scenarios. These may include imposing stricter load limits, restricting certain vehicle classes, or installing weight monitoring systems and warning signage at approach roads to deter excessive loading.

The principal objective of the eradication phase is to eliminate the underlying conditions that resulted in the incident and to implement corrective and preventative measures that ensure continued structural integrity and public safety.

### 5.5. Phase 5—Documentation

The final phase involves systematic documentation of all significant incidents that have occurred over the life cycle of the structure. This includes detailed records of the root causes, containment and eradication measures implemented, and any secondary defects that emerged in other structural components as a consequence of the initial incident. The primary objective is to establish a comprehensive repository of lessons learned, which serves as a reference for future incident response strategies. Thorough documentation enables more efficient resolution of similar issues in other structures by reducing the time required to identify effective solutions. Furthermore, it contributes to the broader knowledge base of potential structural vulnerabilities and failure modes, thereby enhancing hazard awareness and risk mitigation efforts.

## 6. Conclusions

This study presents a comprehensive approach to the assessment, monitoring, and management of a heritage-listed reinforced concrete bridge in Australia. Through detailed field investigations and the deployment of non-invasive sensing technologies, including accelerometers for ambient vibration testing, non-contact laser displacement sensors, and elastic displacement gauges for crack width monitoring, a robust dataset was obtained without compromising the structure’s integrity. These measurements supported the development and validation of a digital twin model, verified through comparison with experimentally identified natural frequencies and mode shapes.

The digital twin was subsequently employed to simulate various “what-if” risk scenarios, enabling proactive evaluation of structural vulnerabilities under different loading and damage conditions. The simulation scenarios considered both the passage of overweight vehicles and the occurrence of scour damage affecting the foundations of one or two bridge piers. The results indicate that the passage of overweight vehicles significantly increases the force demand on the bridge girders, potentially leading to structural damage. These findings reinforce the necessity of strict enforcement of load restrictions on this heritage bridge. Furthermore, simulations of scour-induced foundation degradation revealed notable shifts in natural frequencies and mode shapes, suggesting that such damage can alter the bridge’s dynamic characteristics and may be detectable through ambient vibration monitoring.

Building on these insights, a structured Incident Response Framework (IRF) was proposed to enhance the integration of Structural Health Monitoring (SHM) systems into routine asset management practices. This framework facilitates the timely detection, classification, and mitigation of structural anomalies, thereby improving resilience and extending service life while minimizing intervention costs.

The methodology outlined in this paper demonstrates the practical benefits of combining digital technologies and SHM for the preservation of heritage infrastructure, offering a replicable model for similar bridges worldwide.

## Figures and Tables

**Table 1:** Material properties of St Kilda Street Bridge [26].

Parameters	Values
Concrete strength (beam)	12.4 MPa ^1^
Concrete strength (column)	17.2 MPa ^1^
Concrete Elastic Modulus	22,500 MPa
Corrosion	Low to moderate
Porosity	High
Chloride content	Low

^1^ Values from coring tests.

**Table 2:** Comparison of fundamental frequency.

Field Measurement	FE Model	Difference (%)
20.15 Hz	19.51 Hz	3.2%

**Table 3:** Comparison of structural actions due to the passage of overweight vehicles.

Design Vehicle	M13.5 (AASHTO [32])	T44 (1992 Austroads [33])	M1600 (AS5100.2-2017 [34])
Total vehicle weight	135 kN	432 kN	1440 kN
No. axle	2	5	12
Edge girder-max. shear force (kN)	12.3	20.7	38.1
Edge girder-max. bending moment (kNm)	9.99	18.1	33.5
Interior girder-max. shear force (kN)	48.2	76.1	117.1
Interior girder–max. bending moment (kNm)	34.7	57.1	110.4
Max deflection (mm)	1.3 (*L*/4040)	2.0 (*L*/2613)	3.2 (*L*/1650)

**Table 4:** Fundamental frequencies shift due to loss of foundation to scouring.

No Damage	Scour C1	Scour C1 & D1
19.51 Hz	10.54 Hz	7.91Hz

## Data Availability

The measurement of data from the ambient vibration test is available upon request.
